# Estrogen signaling and senescence-associated secretory phenotype in ovarian cancer: remodeling the tumor immune microenvironment and therapy response

**DOI:** 10.1093/bib/bbaf631.058

**Published:** 2025-12-12

**Authors:** Cheuk Shuen Li, Zhendan Liu, Sike Yu, Qi Liang, Yi Song, Qiuchen Zhao, Lanqi Gong

**Affiliations:** Key Laboratory of Artificial Organs and Computational Medicine in Zhejiang Province, Shulan (Hangzhou) Hospital, China; Hangzhou Institute for Advanced Study, University of Chinese Academy of Sciences, China; Hangzhou Institute for Advanced Study, University of Chinese Academy of Sciences, China; Key Laboratory of Artificial Organs and Computational Medicine in Zhejiang Province, Shulan (Hangzhou) Hospital, China; Key Laboratory of Artificial Organs and Computational Medicine in Zhejiang Province, Shulan (Hangzhou) Hospital, China; Department of Oncology, University of Cambridge, United Kingdom; Department of Clinical Oncology, School of Clinical Medicine, University of Hong Kong, Hong Kong

## Abstract

**Background:**

Estrogen signaling and senescence-associated secretory phenotype (SASP) both exert dual roles in cancer, restraining proliferation but fueling chronic inflammation and immune evasion. In ovarian cancer, how estrogen signaling intersects with SASP to remodel the tumor immune microenvironment and influence therapeutic responses remains poorly understood, but will generate crucial prognostic implications.

**Methods:**

By integrative multi-modal analyses, we identified significant associations between estrogen signaling and SASP activities in the microenvironment. Estrogen receptor ESR1 was predominantly expressed in epithelial cells, where its high expression coincided with robust SASP signatures and correlated with poor survival in TCGA cohorts. Spatial analyses of patients receiving taxane- and platinum-based neoadjuvant chemotherapy (NACT) were performed.

**Results:**

Good responders exhibited markedly reduced SASP signaling, accompanied by an increased macrophage ratio and a reduced epithelial ratio, underscoring a dynamic remodeling of the TIME under therapy. Notably, we uncovered a cycling macrophage population enriched in OC that displayed strong senescence and SASP pathway activation. Ligand–receptor analysis further revealed reciprocal communication together sustaining a pro-tumorigenic and chemoresponse-associated SASP niche.

**Conclusion:**

This finding highlights that the ESR1–SASP axis is not merely a promoter of progression but a critical determinant of therapy response. Our findings thus position it as a pivotal biomarker and a promising therapeutic target.

**References:**

1. Chen I-C., Lin C-H., Chang D-Y., Chen TW-W., Wang M-Y., Ma W-L., Lin Y-T., Huang S-M., Hsu C-L., Lu Y-S. ‘Hormone therapy enhances anti-PD1 efficacy in premenopausal estrogen receptor-positive and HER2-negative advanced breast cancer.’ *Cell Reports Medicine* 2025;6(1):101879.

2. Denisenko E., de Kock L., Tan A., Beasley A.B., Beilin M., Jones M.E., … & Forrest A.R. ‘Spatial transcriptomics reveals discrete tumour microenvironments and autocrine loops within ovarian cancer subclones.’ *Nature Communications* 2024;15(1):2860.

